# Cost-effectiveness of prophylactic ramosetron in the prevention of postoperative nausea and vomiting

**DOI:** 10.1371/journal.pone.0309592

**Published:** 2024-10-17

**Authors:** David Suh, Dong-Won Kim, Seung-Mi Lee, Yu-Seon Jung, Sun-Young Jung, Chul-Min Kim

**Affiliations:** 1 School of Public Health, University of Michigan, Ann Arbor, MI, United States of America; 2 College of Pharmacy, Chung-Ang University, Seoul, Republic of Korea; 3 College of Pharmacy, Daegu Catholic University, Gyeongsan, Republic of Korea; 4 College of Medicine, Catholic University of Korea, Seoul, Republic of Korea; Taipei Medical University, TAIWAN

## Abstract

**Objectives:**

This study was conducted to assess the cost-effectiveness of prophylactic use of ramosetron compared to no antiemetic medications for the prevention of postoperative nausea and vomiting (PONV) from the healthcare payer and societal perspectives in South Korea.

**Method:**

A decision analytic model was constructed to assess the cost-effectiveness of prophylactic ramosetron use versus no antiemetic therapy at 24-hour and 48-hour periods post-surgery over a 5-day duration. The model was populated using costs and utility parameters from published studies as well as from surveys of an expert panel of physicians using structured questionnaires. The cost parameters included the costs of drugs, treatment, patient time, productivity loss, and transportation. Effectiveness was measured using quality adjusted life years (QALYs). The study outcome was the incremental cost-effectiveness ratio (ICER). The parameter uncertainties were addressed using deterministic and probabilistic scenario analyses.

**Results:**

The base-case analysis showed that, on average, patients treated with prophylactic ramosetron had lower costs from both the healthcare payer (US$16.88 vs US$17.33) and societal (US$16.89 vs US$18.72) perspectives and higher QALYs (0.0121 vs 0.0114) over the 5-day study duration compared to patients without any antiemetic medications. Deterministic and probabilistic sensitivity analyses were conducted to examine the robustness of results for the parameters included in the model. The acceptability curve probability showed that treating patients with ramosetron compared to no antiemetic medications was more than 99% cost-effective at a willingness-to pay threshold of US$5,000/QALY from both payer and societal perspectives.

**Conclusion:**

The results demonstrated that prophylactic use of ramosetron compared to no antiemetic therapy is highly cost-effective to prevent PONV for patients undergoing surgery from both healthcare payer and societal perspectives. The cost effectiveness is the result of the decrease in the incidence of PONV and the direct treatment costs of severe PONV with improved patient quality of life.

## Introduction

The prevalence of postoperative nausea and vomiting (PONV) is estimated at 27.7% worldwide and is 1.7 times higher during the first 24-hour postoperative period compared to any other postoperative period [1]. In South Korea, approximately 41% of surgical patients experienced PONV [2]. Based on clinicians’ concerns about undertreating preventable PONV, current guidelines established under organizations such as the American Society of Enhanced Recovery and Society for Ambulatory Anesthesia and the ERAS^®^ Society recommend more aggressive prophylactic antiemetic use to decrease patients’ risk of experiencing PONV [3–5]. Patients who do not receive prophylactic antiemetic medications for PONV can experience impaired mobilization, dehydration, an inability to tolerate fluids and food delivered orally, and an electrolyte imbalance [6, 7]. Patients with clinically significant PONV experienced, on average, a 1-day longer hospital stay compared to those without clinically significant PONV [6, 7]. Thus, PONV is a postoperative outcome that patients would likely want to avoid [8].

Previous studies from the hospital perspective have shown that prophylactic antiemetics were cost-effective in preventing PONV compared to no antiemetics [9, 10]. These studies included costs of medications and materials used for treating PONV, personnel costs (physicians and nurses) associated with treating nausea and vomiting, and costs associated with readmission. In one study among ambulatory gynecology surgery patients in Canada, the incremental cost-effectiveness ratio (ICER) of dolasetron vs. no prophylactic antiemetics was CA$5.82 (US$7.16 in 2024) per additional PONV-free patient [9]. Another study found that use of prophylactic droperidol therapy compared to placebo in high risk ambulatory surgical patients was cost-effective, with an ICER of US$3.40 (US$7.29 in 2024) for 0.625mg of droperidol, and US$2.30 (US$4.93 in 2024) for 1.25mg of droperidol [10].

Ramosetron is a 5-HT3 receptor antagonist mainly used for preventing PONV and has proven its effectiveness over ondansetron for preventing late postoperative nausea, having a comparable adverse event profile with less frequently reported dizziness [11–13].

Although treatment guidelines recommend antiemetic prophylaxis use to prevent PONV, real-world clinical practice variations exist in prophylactic antiemetic regimens in South Korea [14, 15]. To the best of our knowledge, previous studies have not conducted a cost-effectiveness analysis with ramosetron to support health decision-making. This study was conducted to assess the cost-effectiveness of prophylactic ramosetron use compared to no antiemetic medications for the prevention of PONV from the healthcare payer and societal perspectives in South Korea.

## Materials and methods

### Model structure

This cost-effectiveness analysis was performed using a decision analytic model to assess prophylactic ramosetron use compared to no antiemetic therapy in preventing PONV for surgical patients over a 5-day time horizon using Microsoft Excel® (Redmond, WA, USA). The model was assessed from the healthcare payer and societal perspectives in South Korea. In addition to the direct treatment costs included in the payer perspective, the societal perspective included costs of treatment time, wage loss, and transportation.

**Fig 1:**
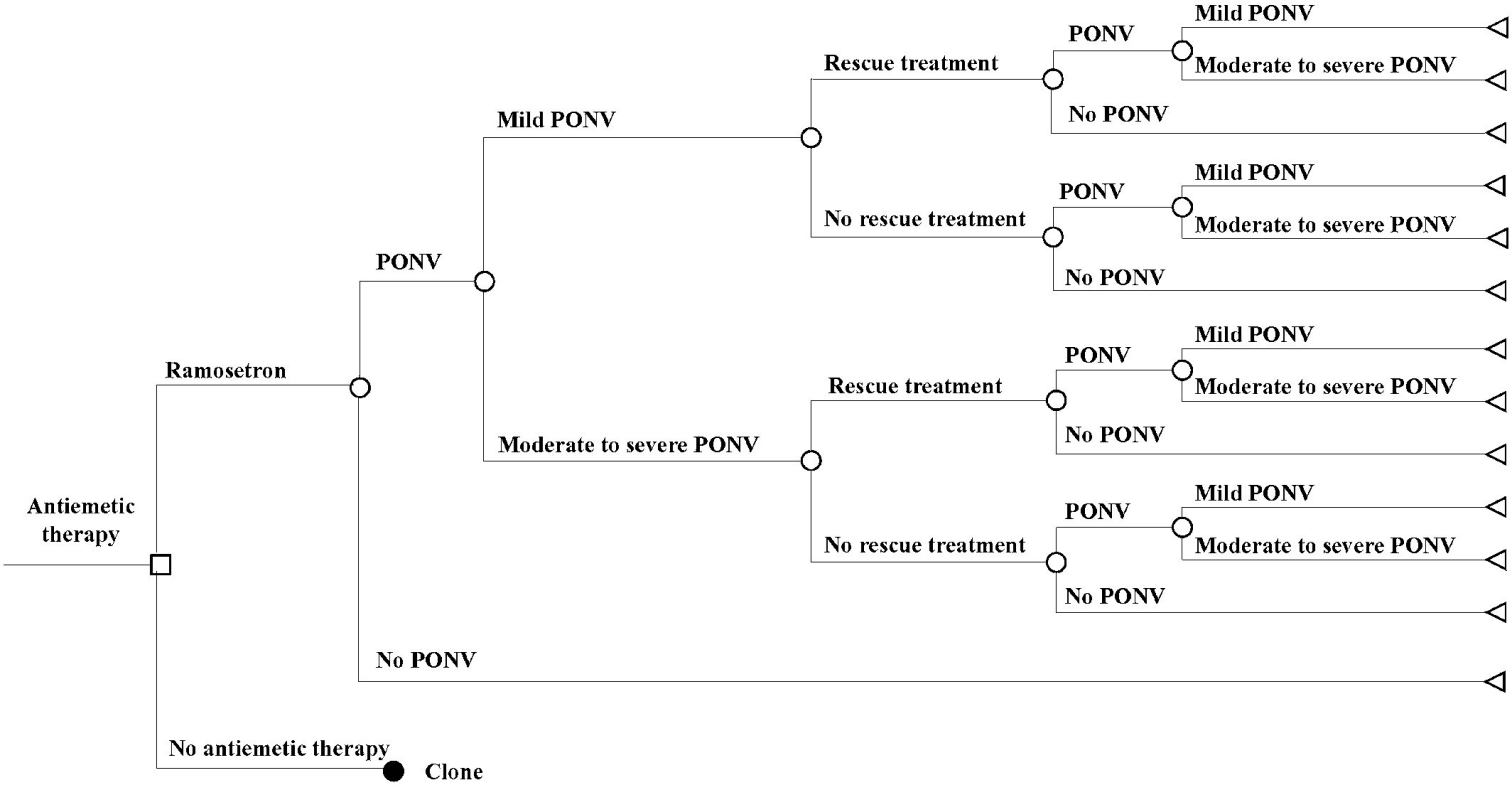
Decision tree model for assessing cost-effectiveness of ramosetron compared to no antiemetic therapy.

A decision tree model was constructed with acute and delayed postoperative phases to estimate and compare the costs and outcomes of prophylactic ramosetron use versus no antiemetics for PONV (Fig 1). The outcomes of the model were no PONV, mild PONV, and moderate-to-severe PONV.

Study patients included those who underwent any surgery, and the PONV severity was estimated based on surveying an expert panel of seven physicians using structured questionnaires. The parameters included in the analytic model are shown in Table 1. Direct treatment costs and patient time and productivity costs from the healthcare payer and the societal perspectives are shown. The total expected costs and quality-adjusted life years (QALYs) that were associated with PONV episodes were calculated over a 5-day time horizon. Costs and outcomes were not discounted due to the short time frame of analyses.

**Table 1:** Model input parameters for the base-case scenario.

Parameters	Value, US$ (KR₩)	Range, US$	Distribution	Source
Costs of drugs				
Ramosetron (0.3mg)	$13.94 (₩19,092)	$13.11–$16.04	Gamma	[16]
Metoclopramide (10mg)	$0.27 (₩375)	$0.25–$0.30	Gamma	[16]
Dexamethasone (5mg)	$1.60 (₩2,190)	$1.36–$1.83	Gamma	[16]
Palonosetron (0.075mg)	$9.43 (₩12,922)	$8.44–$10.42	Gamma	[16]
Costs of treating PONV				
Physician hourly wage	$53.92 (₩73,868)	$48.24–$59.62	Gamma	[17]
Physician time for treating PONV	2 minutes	1.80–2.20	Normal	[18]
Nurse hourly wage	$15.72 (₩21,529)	$14.06–$17.38	Gamma	[17]
Nurse time for treating PONV	14 minutes	12.60–15.40	Normal	[18]
Cost per day of hospitalization due to PONV	$188.29 (₩257,932)	$168.43–$208.15	Gamma	[17, 19]
Additional days of hospitalization due to PONV before discharge	1.67 days	1.39–1.94 days	Normal	Panel survey
Cost per outpatient visit after discharge	$23.22 (₩31,809)	$21.95–$25.67	Gamma	[17, 19]
No. of outpatient visit after discharge	0.67	0.62–0.71	Normal	Panel survey
Time and productivity costs due to PONV				
Patient wage per hour	$13.09 (₩17,931)	$11.71–$14.47	Gamma	[20]
Wage loss per outpatient visit	$32.56 (₩44,599)	$29.13–$36.01	Gamma	[21, 22]
Wage loss per day of hospitalization	$104.72 (₩143,448)	$93.67–$115.76	Gamma	[21, 22]
Cost of transportation per visit	$8.94 (₩12,247)	$8.01–$9.88	Gamma	[23]
Duration of analysis	5 days	4–6 days	Normal	Panel survey
Utility values (mean, SE)				
No PONV	0.90	0.09 (SE)	Beta	[24–27]
Mild PONV	0.70	0.07 (SE)	Beta	
Moderate-to-severe PONV	0.24	0.02 (SE)	Beta	

KR, Korean; PONV, postoperative nausea and vomiting; No, number; CI, confidence interval; SE, standard error

### Modeling time to PONV

A parametric survival model was used to estimate the probabilities of PONV from previous studies that comprised a total pool of 271 patients between the ages of 18–68 who underwent breast or thyroid surgery [28–30]. Ramosetron treatment was associated with a reduced likelihood of moderate-to-severe PONV when measured at the initial 24-hour and 48-hour postoperative intervals. We then constructed a model using the proportion of patients with PONV at the 24-hour and 48-hour postoperative time points. The time to PONV occurrence was fitted to a parametric curve, and the proportion of PONV patients in each treatment option at a given time point was estimated from the areas under the PONV curve [31]. The survival model was extrapolated over the 5-day postoperative period.

The PONV incidence curve was created by fitting the survival function of four parametric distributions (i.e., exponential, Weibull, log-logistic, and log-normal distributions) [32]. The best-fit curve was constructed using the Akaike Information Criterion and the Bayesian Information Criterion goodness-of-fit statistics. The Weibull distribution was selected for both the prophylactic ramosetron and no antiemetic therapy groups based on statistical tests (Table 2). The Weibull distribution is preferred in all conditions when the hazard rate is decreasing, increasing, or constant over time [33]. All statistical analyses were performed using R 4.2.1 programs and the ’Survival’ package was used for the parametric survival function [34, 35].

**Table 2:** Function of time to PONV event for the base-case scenario.

Parameters	Ramosetron	No antiemetics	Distribution	Source
Function of time to PONV				
Intercept	-2.0997	-2.7831	Weibull	[28–30, 41–43]
Log(scale)	0.8475	0.7985	Weibull	[28–30, 41–43]
Proportion of severity in PONV				
0–24 hours after surgery				
Mild PONV	0.7727	0.7273	Beta	Panel survey
Moderate-to-severe PONV	0.2273	0.2727	Beta	Panel survey
24–48 hours after surgery				
Mild PONV	0.7633	0.7018	Beta	Panel survey
Moderate-to-severe PONV	0.2367	0.2982	Beta	Panel survey
Proportion of services used due to PONV				
Additional hospital stay	0.0833	0.1333	Beta	Panel survey
Outpatient visit after discharge	0.0200	0.0200	Beta	Panel survey

PONV, postoperative nausea and vomiting

### Prophylactic antiemetic therapy

Anesthesia and monitoring were assumed to be applicable to all patients. Patients in the ramosetron treatment group received ramosetron (0.3mg) intravenously and the those in the no antiemetic treatment group received saline solution (2ml) intravenously [36]. All patients were postoperatively transferred to the post-anesthesia care unit. Almost all patients were extubated and observed in the post-anesthetic care unit for approximately 1 or 2 hours before being transferred to the general ward of the hospital.

### Resource utilization and costs

Direct treatment costs over the 5-day postoperative period from the payer perspective included costs of the antiemetic drugs, rescue medications (e.g., metoclopramide, dexamethasone, and palonosetron), treating adverse effects associated with PONV, additional hospitalization due to PONV, outpatient visits after discharge, and costs associated with physicians’ and nurses’ time for treating PONV, which were calculated based on hourly wages for the time spent treating patients with PONV during hospitalization [17–19, 25, 37].

The unit costs of medications, including ramosetron, were obtained from the Korean Health Insurance Review & Assessment Service (HIRA) based on costs as of December 2023 [16]. Costs of rescue treatments were estimated using a structured questionnaire to survey an expert panel of seven surgeons at university hospitals. The anesthesia and postoperative care costs were assumed to be the same for both groups.

Costs calculated from the societal perspective included costs for work productivity loss (i.e., lost or impaired ability to work due to PONV), patients’ time associated with outpatient visits due to PONV after discharge, and transportation for outpatient visits [20–23]. While caregiver related costs are commonly included for the societal perspective, this study did not separately include caregiver related costs because PONV occurring in the hospital was taken care of by nurses; thus, we included nurse costs associated with treating PONV. All included costs associated with PONV are listed in Table 1 and were adjusted to 2024 values using the Korean medical price index. These costs in Korean Won (KR₩) were converted to the United States dollar (US$) equivalent by using the average currency exchange rate of KR₩1 = US$0.00073 from January 1 to March 30, 2024.

### Rates of adverse drug events and associated treatment costs

Adverse events that required rescue medications for treating PONV included headache, dizziness, and drowsiness [12, 15]. The treatment costs associated with these adverse drug events were applied based on inpatient services rendered. The costs of treating adverse drug events were calculated by applying the rate of adverse events in patients with PONV receiving antiemetics to the subsequently utilized resources. The incidence of adverse drug events and resources used were based on responses to a structured questionnaire by an expert panel of seven surgeons at university hospitals.

### Health utility of PONV

The utility values for patients who experienced mild or moderate-to-severe PONV were extracted from published studies that elicited utility values for health states associated with the level of nausea and vomiting in patients who received chemotherapy [24, 26, 27, 38–40]. Based on the results of these studies, the classified health states in the present study model included: complete protection due to antiemetic drugs (i.e., the health state of no emesis, no rescue therapy, and a maximum nausea visual analog scale score <25 mm on a 100-mm scale), complete response to antiemetic drugs (i.e., the health state of no emesis, no rescue therapy, and a maximum nausea visual analog scale score ≥25 mm on a 100-mm scale), and incomplete response to antiemetics, with utility values of 0.9, 0.7, and 0.2, respectively. The present study assumed that health states associated with nausea and vomiting would not differ among diseases (e.g., cancer vs. other diseases requiring surgery) and that the health states of no PONV, mild PONV, and moderate-to-severe PONV would correspond to complete protection, complete response, and incomplete response, respectively (Table 1).

The utility values were assigned according to the estimated curve of PONV over a 5-day period. The proportion of patients with mild and moderate-to-severe PONV was based on surveying an expert panel of seven surgeons using a structured questionnaire. As costs and outcomes were tracked over a 5-day time horizon in the model, the QALYs for the same period were calculated as follows [26, 39]: QALYs for 5 days = (Utility at Day 1 acute phase + Utility for Days 2–4 delayed phase) / 365.25.

### Base-case analysis

The primary outcomes were the expected costs and expected utilities with ramosetron use versus no antiemetic medication use. These costs and utilities were then used to calculate the ICER, which was calculated by dividing the difference in costs by the differences in QALYs between the two treatment options (ramosetron versus no antiemetics).

### Sensitivity analyses

One-way and probabilistic sensitivity analyses were performed to assess the uncertainty in the decision analytic model. One-way sensitivity analyses were performed to assess the impact of each parameter on the ICER by varying the value of the inputs within a reasonable range as presented in Table 1. Input parameters were varied based on their respective 95% confidence intervals from prior data or a +/- 10% range in the parameters.

Results of the one-way sensitivity analyses are presented in a tornado diagram. The tornado diagram evaluated the impact of key parameters on ICER values rather than using fixed willingness to pay thresholds because HIRA, which is the Korean government agency that evaluates the cost-effectiveness of drugs, uses a wide range of willingness to pay thresholds depending on factors such as the therapeutic class of medication.

Probabilistic sensitivity analyses with 1000 iterations of a Monte Carlo simulation were performed to assess the robustness of the cost-effectiveness results. These analyses were conducted by assigning an appropriate distribution to each input parameter (i.e., normal distribution for the number of outpatient visits, beta distribution for transition probability, and gamma distribution for costs and utility values) [32]. Results of the probabilistic sensitivity analyses were presented in a cost-effectiveness plane and a cost-effectiveness acceptability curve in the results section.

### Ethics statement

This modeling study did not involve any patients or human subjects, and according to the Institutional Review Board of Korea Institute for Bioethics Policy, the requirement of ethical approval was not applicable for this study.

## Results

The curve depicting the function of time on the incidence of PONV, the proportion of patients with PONV at each timepoint, and the proportion of patients who had additional days of hospitalization due to PONV is presented in Table 2. Ramosetron-based prophylactic treatment was associated with a reduced likelihood of moderate-to-severe PONV when measured at the first 24-hour and 48-hour postoperative intervals. The proportion of patients who required an additional hospital stay due to PONV was 0.0833 in the ramosetron group and 0.1333 in the no antiemetic group.

The cost-effectiveness results of the base-case analysis from the payer perspective are shown in Table 3 and from the societal perspective are presented in Table 4. The overall treatment costs of the ramosetron group were lower than those of the no antiemetic group from both the healthcare payer (US$16.88 vs US$17.33) and societal (US$16.89 vs US$18.72) perspectives. The QALYs gained over the 5-day period were 0.0121 with ramosetron and 0.0114 without antiemetic therapy.

**Table 3:** Cost-effectiveness of prophylactic ramosetron compared to no antiemetic therapy from the healthcare payer perspective.

Parameters	Ramosetron	No antiemetics	Incremental analysis	
	US$ (KR₩)	US$ (KR₩)	US$	(KR₩)
Direct costs treating PONV				
Prophylactic drugs	$13.94 (₩19,092)	$ 0.00 (₩0)	$13.94	(₩19,092)
Rescue medications	$1.69 (₩2,315)	$7.84 (₩10,737)	−$6.15	(−₩8,421)
Physicians’/nurses’ effort	$1.18 (₩1,615)	$5.57 (₩7,626)	−$4.39	(−₩6,011)
Additional hospitalization	$0.07 (₩102)	$3.92 (₩5,372)	−$3.85	(−₩5,270)
Outpatient visit AD	$0.00 (₩0)	$0.01 (₩8)	−$0.00	(−₩8)
Total costs from payer perspective	$16.88 (₩23,125)	$17.33 (₩23,735)	−$0.45	(−₩610)
(95% CI)	($14.26 to $19.85)	($15.48 to $19.33)	(−$3.21 to $2.67)	(−₩4,396 to ₩3,653)
Quality Adjusted Life Years	0.0121	0.0114	0.0007	0.0007
(95% CI)	(0.0121 to 0.0122)	(0.0111 to 0.0117)	(0.0005 to 0.0010)	(0.0005 to 0.0010)
ICER from payer perspective			−$1,080.88/QALY	(−₩1,403,735/QALY)
(95% CI)			−$5,311.32 to $3,642.62	(−₩6,897,812 to ₩4,730,680)

KR, Korean; PONV, postoperative nausea and vomiting; AD, after discharge; CI, confidence interval; ICER, incremental cost-effectiveness ratio.

**Table 4:** Cost-effectiveness of prophylactic ramosetron compared to no antiemetic therapy from the societal perspective.

Parameters	Ramosetron	No antiemetics	Incremental analysis	
	US$ (KR₩)	US$ (KR₩)	US$	(KR₩)
Direct costs treating PONV				
Prophylactic drugs	$13.94 (₩19,092)	$ 0.00 (₩0)	$13.94	(₩19,092)
Rescue medications	$1.69 (₩2,315)	$7.84 (₩10,737)	−$6.15	(−₩8,421)
Physicians’/nurses’ effort	$1.18 (₩1,615)	$5.57 (₩7,626)	−$4.39	(−₩6,011)
Additional hospitalization	$0.07 (₩102)	$3.92 (₩5,372)	−$3.85	(−₩5,270)
Outpatient visit AD	$0.00 (₩0)	$0.01 (₩8)	−$0.00	(−₩8)
Time and productivity costs				
Transportation for outpatient visit AD	$0.00 (₩0)	$0.00 (₩2)	−$0.00	(−₩2)
Patient time for outpatient visit AD	$0.00 (₩0)	$0.02 (₩22)	−$0.02	(−₩22)
Productivity loss	$0.01 (₩17)	$1.37 (₩1,883)	−$1.36	(−₩1,866)
Total costs from societal perspective	$16.89 (₩23,142)	$18.72 (₩25,642)	−$1.83	(−₩2,508)
(95% CI)	($14.27 to $19.87)	($16.87 to $20.72)	(−$4.59 to $1.28)	(−₩6,293 to ₩1,753)
Quality Adjusted Life Years	0.0121	0.0114	0.0007	0.0007
(95% CI)	(0.0121 to 0.0122)	(0.0111 to 0.0117)	(0.0005 to 0.0010)	(0.0005 to 0.0010)
ICER from societal perspective			−$2,615.68/QALY	(−₩3,583,126/QALY)
(95% CI)			(−$7,045.86 to $1,820.78)	(−₩9,651,869 to ₩2,494,223)

KR, Korean; PONV, postoperative nausea and vomiting; AD, after discharge; CI, confidence interval; ICER, incremental cost-effectiveness ratio.

Decreased treatment costs with QALY gains resulted in the treatment of patients with ramosetron being a cost-effective therapy compared to no antiemetic prophylaxis, from both the healthcare payer and societal perspectives.

**Fig 2:**
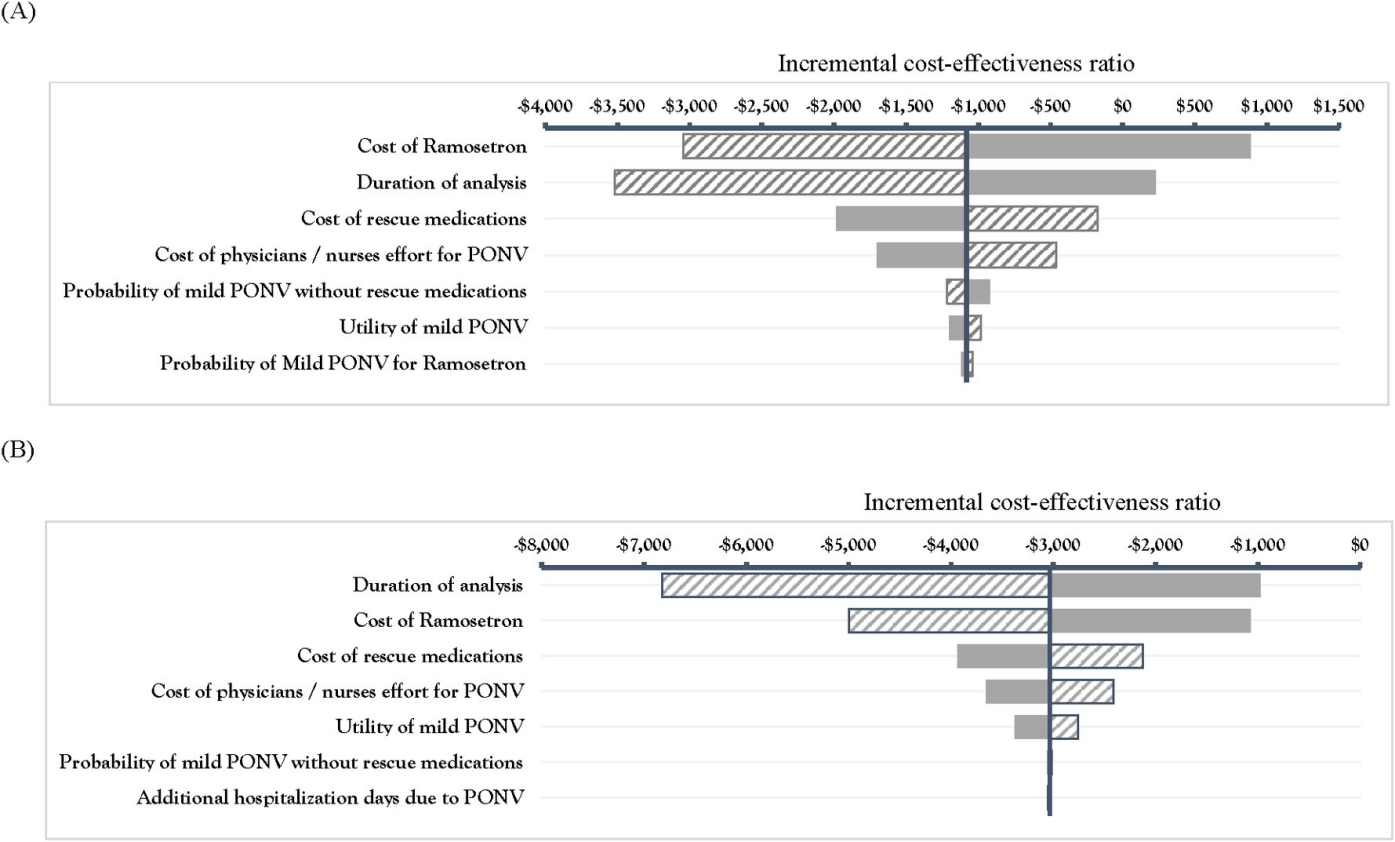
Tornado diagram of one-way sensitivity analyses for ramosetron compared to no antiemetic therapy. (A) Sensitivity analysis from the healthcare payer perspective; (B) Sensitivity analysis from the societal perspective.

The tornado diagram showed that the ICER value from the payer’s perspective was influenced most by the cost of ramosetron, followed by the duration of analysis, the cost of rescue medications, and the cost of physicians’/nurses’ efforts for treating PONV (Fig 2). The ICER value from the societal perspective was influenced in order by the duration of analysis, the cost of ramosetron, and the cost of rescue medications. The results of one-way sensitivity analyses showed some variations in the order of the influential parameters based on the perspective chosen, although the most influential parameters were the same regardless of perspective.

The application of low and high estimates for the cost of ramosetron resulted in an ICER range of US$−3,050 to US$800/QALY. The negative ICER values resulted from decreased costs with QALY gains from prophylactic ramosetron use. The results of one-way sensitivity analyses showed some variations in the order of the influential parameters based on the perspective chosen.

**Fig 3:**
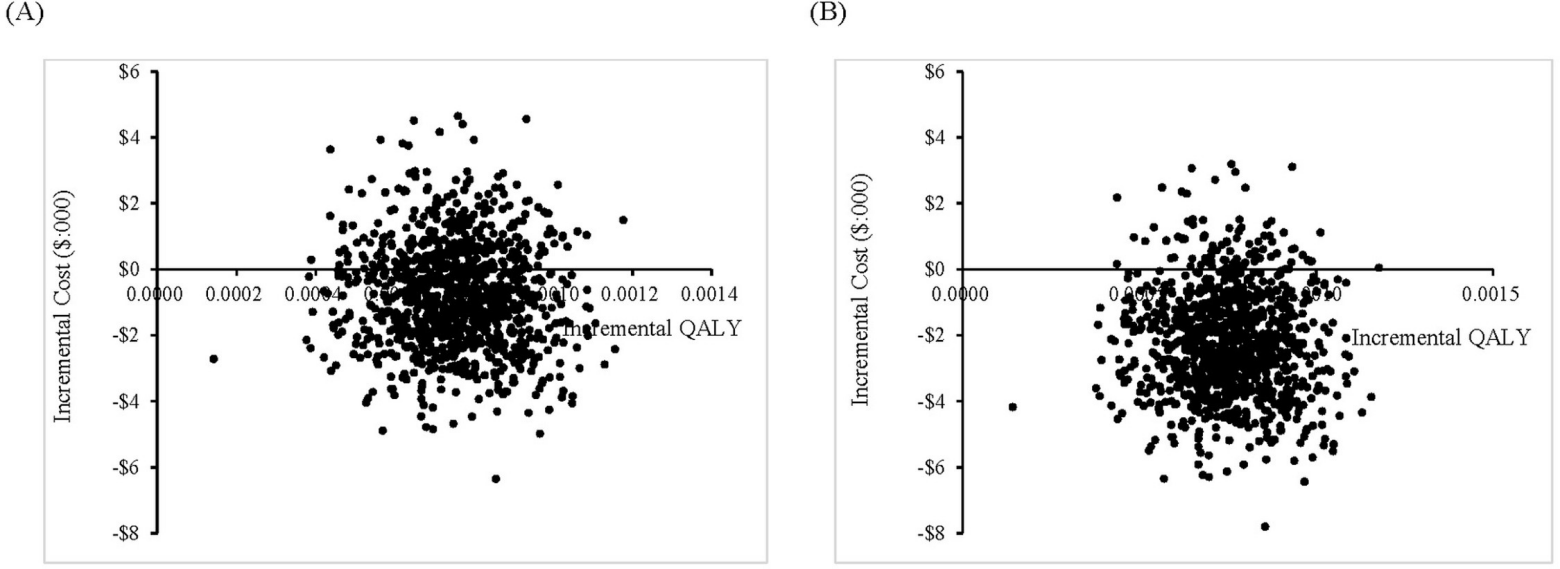
Cost-effectiveness scatterplot of ramosetron compared to no antiemetic therapy. (A) From the healthcare payer perspective; (B) From the societal perspective.

The results of probabilistic sensitivity analyses conducted through Monte Carlo simulation are displayed as a scatterplot, with all 1,000 iterations resulting in increased QALYs with ramosetron use (Fig 3).

**Fig 4:**
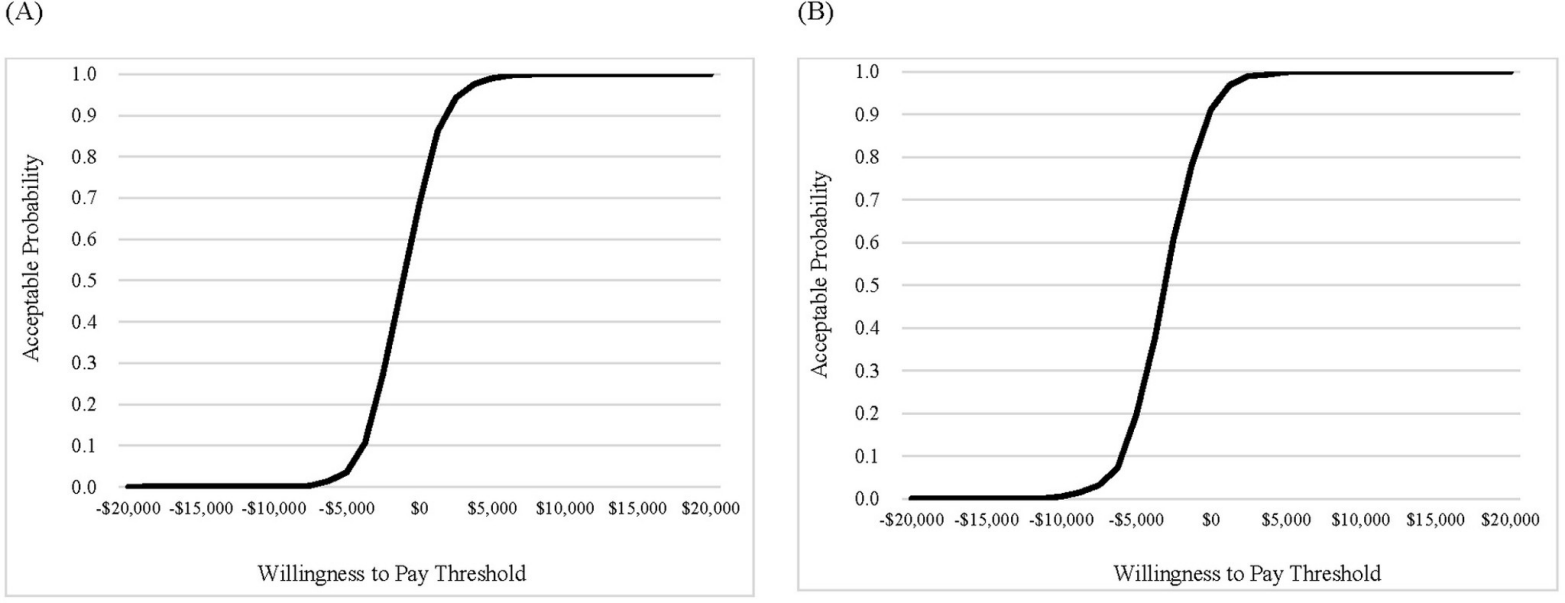
Cost-effectiveness acceptability curves of ramosetron compared to no antiemetic therapy. (A) From the healthcare payer perspective; (B) From the societal perspective.

Probabilities of calculated ICER values being accepted at various willingness-to-pay thresholds, ranging from −US$20,000 to US$20,000/QALY, are plotted in Fig 4. From both payer and societal perspectives, the probability that treating patients with ramosetron will be accepted as a cost-effective therapy compared to no antiemetic medications is more than 99% at a threshold of US$5,000/QALY. This threshold value is much lower than commonly used thresholds of US$18,250/QALY in Korea and US$50,000/QALY in the US [44, 45].

## Discussion

To the best of our knowledge, this is the first study to assess the cost-effectiveness using a decision tree model of ramosetron vs. no antiemetic therapy after surgery in South Korea. Based on this study’s findings from both the healthcare payer and societal perspectives, prophylactic ramosetron use is a cost-effective strategy compared to no antiemetic therapy for preventing PONV in South Korea. The ICER of ramosetron versus no antiemetic therapy was −US$636/QALY from the payer perspective and −US$2,616/QALY from the societal perspective; these values demonstrate that prophylactic use of ramosetron produces cost-savings compared to no antiemetic therapy.

Few previous cost-effectiveness assessments of antiemetic prophylaxis of PONV have been conducted; however, the outcomes measured in these studies varied widely and were thus difficult to compare with our study based on a common outcome measurement [9, 10]. Although these previous studies used different outcome parameters, the studies also showed that prophylactic antiemetic therapy was cost-effective compared to no prophylactic therapy. These studies included direct medical costs such as drug costs of prophylactic and rescue therapies, and other costs such as treating adverse reactions, nursing labor costs associated with PONV, and unplanned readmission due to PONV.

In our study, the primary factors contributing to the cost savings associated with prophylactic ramosetron were lower costs from shorter hospitalizations and decreased time costs of physicians’/nurses’ effort treating PONV. These findings were consistent with those of previous studies: the incremental nursing time associated with treating PONV was found to be, on average, 14 minutes longer (68 minutes in patients without PONV vs. 82 minutes in patients with PONV), which resulted in an adjusted incremental cost of US$ 75 (US$ 110.83 in 2024) per patient [18]. Patients who experienced PONV had longer stays in the post-anesthesia care unit (by 25 minutes on average) than patients without PONV [46]. Another study concluded based on review of patient medical charts that 1.57% of patients who underwent surgery were found to be re-admitted to the hospital due to nausea and vomiting [47]. When taking into account recovery time, the cost-effectiveness of prophylactic ramosetron may be even more pronounced but this study did not take into account postoperative care costs because data were not available [18].

Furthermore, preventing PONV holds significant clinical importance, as more severe instances of PONV deteriorate quality of life, especially in the domains of mobility, self-care, and activity as measured by the EQ-5D [48]. Since mild to severe cases of PONV require additional hospitalization, cost-effectiveness analyses should incorporate the associated utility values; while our study approach includes utilities, previous studies did not take this approach [9, 10, 49].

The acceptable ICER for new drugs depends on country-specific willingness-to-pay thresholds, which is impacted by various factors such as drug innovativeness, the existence of alternative drugs, the disease severity, patients’ unmet needs, and social economic burden. Although there is no explicit acceptable threshold established by those countries that required cost-effective data for use in their decision making, the commonly used thresholds by such countries ranged from US$22,716 to $80,549 [44, 50]. While HIRA did not disclose any specific ICER value when evaluating new drugs, the commonly applied value was below US$18,250 (KR₩25,000,000) per QALY gained for most drugs except for cancer and orphan drugs. HIRA recently reported that the actual accepted median ICER value was US$12,534 (KR₩17,170,000) for HIRA’s drug evaluations except for cancer and orphan drugs in South Korea during 2014–2021 [51]. At this median ICER threshold value, the probability of accepting ramosetron as a cost-effective therapy was more than 99% from both healthcare payer and societal perspectives.

Prior studies have measured the willingness-to-pay amounts for a therapy to prevent or reduce postoperative emesis. Those studies showed that patients place a high value on avoiding PONV, but there is often a lack of consistency in determining the amount patients are willing to pay to prevent PONV because of factors such as patients’ age, the severity and duration of the PONV, previous PONV experiences, and socioeconomic factors [10, 52]. One study found that the median willingness-to-pay amount for a reduction in postoperative emesis among parents of children who underwent surgery was £50 (US$111.86 in 2024), with a range from £5 to £100 or more (US$11.29–223.72 in 2024) [53].

The present study has some limitations that should be considered when interpreting this study’s results. First, due to the short follow-up period in the trial, inherent uncertainty can be a factor in the long-term extrapolation of PONV beyond a 5-day postoperative period, which could subsequently lead to an underestimation of the cost-effectiveness of ramosetron. In addition, there is some inherent uncertainty around the base-case analyses and the resulting ICERs; however, the ICER values are not only well below the commonly used and acceptable willingness-to-pay thresholds but also robust based on the results of the sensitivity analyses. Another limitation is related to the utility values because this study used data available in published literature. Utility values for PONV, which were extracted from previous studies that were conducted in foreign countries, were not directly convertible to a Korean population because validated algorithms were not available. Thus, sensitivity analyses were performed to address the uncertainties associated with utility values. In addition, this study found that the magnitudes of effects of utility values on the results were minor. As there is a lack of cost-effectiveness studies of prophylactic ramosetron use or any other antiemetics for PONV, this present study can provide a framework for assessing the cost-effectiveness of antiemetic use for PONV.

## Conclusions

This study demonstrated that prophylactic ramosetron use is highly cost-effective compared to no antiemetic therapy in patients undergoing surgery from both the healthcare payer and societal perspectives. The cost reduction is largely attributable to the decrease in the incidence of PONV and the direct treatment costs of severe PONV. The findings of this study could be helpful to inform healthcare professionals and policymakers about cost-effective strategies to prevent or reduce the incidence of PONV, which will improve patients’ quality of life and subsequently lead to lower treatment costs. Further research using globally collected real-world data is recommended to facilitate the development of cost-effective treatments and measure the economic burden for the prevention of PONV.
